# Eye-Tracking Based Attention Bias Modification (ET-ABM) Facilitates Disengagement from Negative Stimuli in Dysphoric Individuals

**DOI:** 10.1007/s10608-018-9889-6

**Published:** 2018-01-16

**Authors:** Martin Möbius, Gina R. A. Ferrari, Robin van den Bergh, Eni S. Becker, Mike Rinck

**Affiliations:** 1 Behavioural Science Institute, Radboud University Nijmegen, PO Box 9104, 6500 HE Nijmegen, The Netherlands; 2 Pro Persona, Center for Mental Health Care, Nijmegen, The Netherlands

**Keywords:** Depression, Attention bias modification, Attentional disengagement, Eye-tracking

## Abstract

To address shortcomings of purely reaction-time based attention bias modification (ABM) paradigms, a novel eye-tracking based ABM training (ET-ABM) was developed. This training targets the late disengagement from negative stimuli and the lack of attention for positive information, which are characteristics of depression. In the present study, 75 dysphoric students (BDI ≥ 9) were randomly assigned to either this positive training (PT), or a sham-training (ST) that did not train any valence-specific gaze pattern (positive and negative pictures had to be disengaged from and attended to equally often). Results showed that the PT induced a positive attentional bias (longer fixations of positive than negative pictures). Although the ST group showed an increase in positive attentional bias as well, this increase was not as strong as in the PT group. Compared to the ST, the PT specifically induced faster disengagement from negative pictures. No differential training effects were found on stress responses or state rumination. These results show that the ET-ABM successfully modifies attentional processes, specifically late disengagement from negative stimuli, in dysphoric students, and hence might be a promising alternative to existing ABM paradigms.

## Introduction

With more than 300 million people suffering from depression worldwide, it is amongst the most prevalent mental disorders and, according to the World Health Organization (WHO 2017), has become the leading cause of disabilities, with a major contribution to the overall global disease burden. Despite the variety of existing treatment options for depression, relapse rates are high (e.g., about 30% as reported in Seemüller et al. 2014), with an increasing risk of recurrent depression with each subsequent depressive episode (Steinert et al. 2014). This suggests that the underlying mechanisms which maintain depression are not very well understood yet, and hence may not be targeted sufficiently by current treatment programs.

A potential cognitive vulnerability factor which is thought to maintain depressive symptoms and predispose individuals to repeatedly develop new episodes, is the heightened attention to depression-relevant, negative information compared to positive information, commonly found in depressed individuals (e.g., Armstrong and Olatunji 2012). Research making use of eye-tracking technology suggests that in depression, this so-called negative attentional bias is specifically characterized by difficulties with disengaging attention from negative stimuli once they have become the focus of attention (Sanchez et al. 2013). At the same time, depressed individuals show reduced maintained attention to positive stimuli, compared to healthy individuals (e.g., Ellis et al. 2010; Kellough et al. 2008; Sears et al. 2010).

According to cognitive theories of depression, attentional biases play a causal role in both development and maintenance of this disorder (Beck 1976; Teasdale 1988). As a consequence, computerized training paradigms have been developed, aiming at reducing depressive symptoms through altering maladaptive information processing tendencies; the so-called cognitive bias modification techniques (CBM; Mathews and MacLeod 2005). The most frequently used paradigm for modifying (and measuring) attentional processes is the dot-probe task (MacLeod et al. 2002). On each trial of this task, two stimuli are presented simultaneously on the computer screen, usually a negative picture and a positive (or neutral) picture (or word). After a short delay, both stimuli disappear and a target replaces one of the two stimuli, which participants have to react to. Faster reactions to targets replacing negative compared to positive stimuli indicate a negative attentional bias. In the training version of this task, the targets replace mostly the positive (or neutral) stimuli, such that participants’ attention is selectively trained away from the negative stimuli.

To date, the dot-probe task has mainly been used to assess and modify attentional bias in anxious populations (for review see Cisler and Koster 2010). Only a few studies have tried to modify attentional processes in depressed individuals, with inconsistent findings so far. While some studies managed to reduce the bias toward negative stimuli and accordingly the depressive symptoms, many other studies failed to modify an attentional bias and to replicate the beneficial therapeutic effects on depressed mood or symptoms, questioning the efficacy of attentional bias modification (ABM) procedures for depression (for reviews see Cristea et al. 2015; Mogoaşe et al. 2014). In a paper reflecting on the increasing number of ABM failures Clarke et al. (2014), however, cautioned against taking the absence of evidence in some studies as evidence against the theoretical basis of ABM in general. The authors drew attention to the fact that most studies that succeeded in modifying an attentional bias also induced emotional change. By contrast, those studies that failed to modify an attentional bias also failed to find beneficial effects on mood. This suggests that conventional ABM paradigms may not be optimal for reliably modifying and measuring attentional bias, and that more research is needed into the task conditions under which ABM actually changes attentional processes. According to this argument, more promising clinical applications of ABM depend on the development of more effective attention modification procedures.

One of the most frequently mentioned limitations of the dot-probe and other reaction time (RT) based ABM paradigms is the low reliability of these tasks (e.g., Schmukle 2005; Waechter and Stolz 2015). More importantly, however, the suitability of the dot-probe task has especially been doubted in the context of depression, as it is not clear which component of attention is measured and trained (Leyman et al. 2007). It has been argued that with the longer stimulus durations commonly used in depressed populations (Beevers et al. 2015; Wells and Beevers 2010), participants may shift their attention back and forth between stimuli before the target appears. This in turn leaves undetected whether the task is tapping into heightened vigilance for negative stimuli or the depression-characteristic slowed disengagement from negative stimuli (for a more thorough discussion of the limitations of the dot-probe task, see Ferrari et al. 2016).

Based on the limitations of the dot-probe task and other RT-based ABM paradigms, Ferrari et al. (2016) developed a new ABM paradigm, incorporating eye-tracking technology. This eye-tracking based attentional bias modification (ET-ABM) paradigm allows for the continuous assessment of eye-movements, and hence for a potentially more reliable assessment and training of the specific attentional components that are biased in depression. During this ET-ABM task, participants are trained to disengage their attention from negative pictures and to keep their attention on positive pictures. On each trial of the task, participants first have to fixate a cross on a computer screen, after which two positively and two negatively valenced pictures are presented in a 2 × 2 grid. Importantly, the trial only continues after a sufficiently long fixation of a positive picture (1000 ms). Hence, if a negative picture replaces the cross, the trial only continues when participants look away from the negative picture and fixate one of the positive pictures. If a positive picture replaces the cross, the pictures disappear when participants keep their attention on the fixated positive picture or fixate the other positive picture. As soon as a positive picture has been fixated for 1000 ms, all pictures disappear and a target stimulus is presented at the location of the last fixated picture. The target has to be identified by pressing a corresponding button. Different from previous RT-based ABM paradigms, the training trials in this task only continue if participants show the required viewing pattern, tailoring the pace of the task to the individuals’ performance.

In a first proof-of principle study (Ferrari et al. 2016) with an unselected student sample, this positive training was compared to a negative training with the opposite training contingencies. Thus, participants were trained to direct their gaze to negatively valenced pictures. Results of this first study showed that this ET-ABM training is suited to alter attentional processes relevant in depression: The positive training induced a positive sustained attention bias, that is, longer sustained attention to positive than to negative pictures. More specifically, it trained participants to more quickly disengage their attention from negative stimuli and direct their attention to positive pictures. No such changes were found in the negative training group. Although the training affected mood directly, with the negative group showing a stronger increase in negative mood in response to the training than the positive group, the training did not differentially affect emotional reactions to a subsequent laboratory stressor.

Although this first study provides promising evidence that depression-relevant attentional processes can be altered by means of this novel ABM paradigm, several questions remain to be answered. First, in the previous study, the positive training was compared to a negative training in order to maximize group differences, hence it remains to be established whether the positive training is also superior to a placebo control condition in changing the attentional processes. Second, before applying this training in clinically depressed populations, it should first be tested whether the results can be replicated in a sample with subclinical levels of depression, which is assumed to show a stronger pre-existing negative attentional bias. Finally, training effects in the first study were only tested with the same set of stimuli as used during the training, leaving it open whether training effects are restricted to the specific stimuli being used, or whether the attentional processing of positive and negative stimuli in general is affected.

Hence, the primary aim of this study was to replicate the findings of the previous study in an emotionally vulnerable sample, with a sham training as control condition and different stimulus sets in training and assessment. To this end, we randomly assigned participants with elevated depression scores to one of two training conditions. Half of the participants received the positive training (PT) in which they were trained to direct their gaze away from negative pictures and towards positive pictures. The other half received a sham training (ST), where no valence-specific gaze patterns were reinforced. Importantly, at the beginning of the experiment, a negative mood was induced by means of a sad movie. This allows for re-activation of otherwise latent depressogenic structures in emotionally vulnerable individuals (e.g., Beck 1967) and hence may serve to elicit a negative attentional bias in our sample (for a detailed description of the procedure, see Scher et al. 2005). We expected that, compared to participants in the ST, (1) participants in the PT would show an increase in positive attentional bias (i.e., relatively longer fixations on positive than on negative pictures), and that (2) participants in the PT would specifically learn to faster disengage their attention from negative pictures.

To get a first indication of the potential therapeutic effects of the training, we additionally explored participants’ mood reactivity and recovery in response to a laboratory stressor at the end of the experiment. In their eye-tracking experiment, Sanchez et al. (2013) showed that specifically the slowed disengagement from negative information is related to impaired mood recovery after stress in depressed individuals. In accordance with these findings, we expected that participants in the PT would show better stress recovery than participants in the ST.

## Methods

### Participants

Eighty-four female and 12 male undergraduate students (mean age = 21.67, *SD* = 4.66), of Radboud University Nijmegen, the Netherlands, with elevated depression scores on Beck’s Depression Inventory (BDI-II; Beck et al. 1996; *M* = 15.57, *SD* = 7.13) participated in return for course credit or 20 €. In order to test participants with at least mild depressive symptoms, we only invited students with BDI-II scores higher than 8, which is in line with previous studies (Mastikhina and Dobson 2017; Wells and Beevers 2010). This low cutoff score was chosen to maximize sensitivity of the BDI-II (Sprinkle et al. 2002). Of 841 students who signed up, 96 were invited to the experiment and participated. They were randomly allocated to either the PT (*n* = 50) or the ST (*n* = 46).

### Instruments and Materials

#### Baseline Questionnaires

All of the following questionnaires were administered in the participants’ dominant language (i.e., German or Dutch). Depression levels were assessed with the revised version of Beck’s Depression Inventory (BDI-II, Beck et al. 1996). The internal consistency of the BDI in the current sample was excellent (*α* = .97). To be able to control for possible baseline differences in trait anxiety, the trait subscale of the State-Trait Anxiety Inventory (STAI-T; Spielberger 1989) was administered. The internal consistency of the STAI was excellent (*α* = .97). Moreover, individual differences in ruminative thinking were assessed with the Ruminative Response Scale (RRS; Treynor et al. 2003). As the German and the Dutch version slightly differ in the number of items, we calculated a mean score instead of a sum score. Both, the Dutch and the German version of the RRS showed a good internal consistency (Dutch: *α* = .89; German: *α* = .86).

#### Mood State

Throughout the experiment, participants were asked to rate their current general mood state (i.e., “How is your mood at this moment?”) on a 100 mm Visual Analogue Scale (VAS), ranging from 0 (*very bad*) to 100 (*very good*). In order to assess changes in mood state in response to the stress task, we additionally presented Likert scales as used by Sanchez et al. (2013). Each Likert scale measured mood with three items: happy mood (happy, optimistic, joyful), anxious mood (nervous, tense, anxious), and sad mood (depressed, upset, sad). All items were rated on a scale ranging from 0 (*not at all*) to 10 (*very much*). One extra item was added to assess the tiredness of participants (0 = *not at all*; 10 = *very much*).

#### State Rumination

To assess state rumination in response to the stress task, the Momentary Ruminative Self-focus Inventory (MRSI; Mor et al. 2013) was administered. The MRSI contains six items, measuring momentary self-focused rumination (e.g., “Right now, I am thinking about the possible meaning of the way I feel”). Items are rated on a 7-point scale, ranging from “totally not agree” to “totally agree”.

#### Negative Mood Induction

Negative mood was induced by means of two sequences (i.e., 20 min in total) of the movie “Sophie’s choice”. The two sequences have been shown to effectively induce negative mood in previous studies (e.g., Randall and Cox 2001), and they have been used before to elicit a negative mood state in healthy and dysphoric individuals prior to CBM training (Becker et al. 2016).

### ET-ABM Task

#### Stimuli

In line with the study of Ferrari et al. (2016), a broad range of disorder-nonspecific picture stimuli representing different categories (e.g., people, animals, objects; Nencki Affective Picture System; Marchewka et al. 2014) was selected. Ninety positive and ninety negative pictures (14.3 cm × 10.7 cm) were used during the training phase. A further set of 90 positive and 90 negative pictures was used exclusively during the assessment phases, to allow for testing generalization of training effects to untrained pictures.

For each phase, training and assessment, 45 picture sets were created, always containing two positive and two negative pictures matched on content. The pictures were arranged in a 2 × 2 grid, which separated the screen into four quadrants. The location at which each picture appeared (upper/lower and left/right part of the grid) was counterbalanced across trials. The stimuli were displayed on a black 53.2 cm × 29.9 cm computer screen (BenQ XL2420Z), with 1 cm distance between the pictures. Participants were seated about 60 cm away from the screens’ center.

#### Task Design

**Fig. 1:**
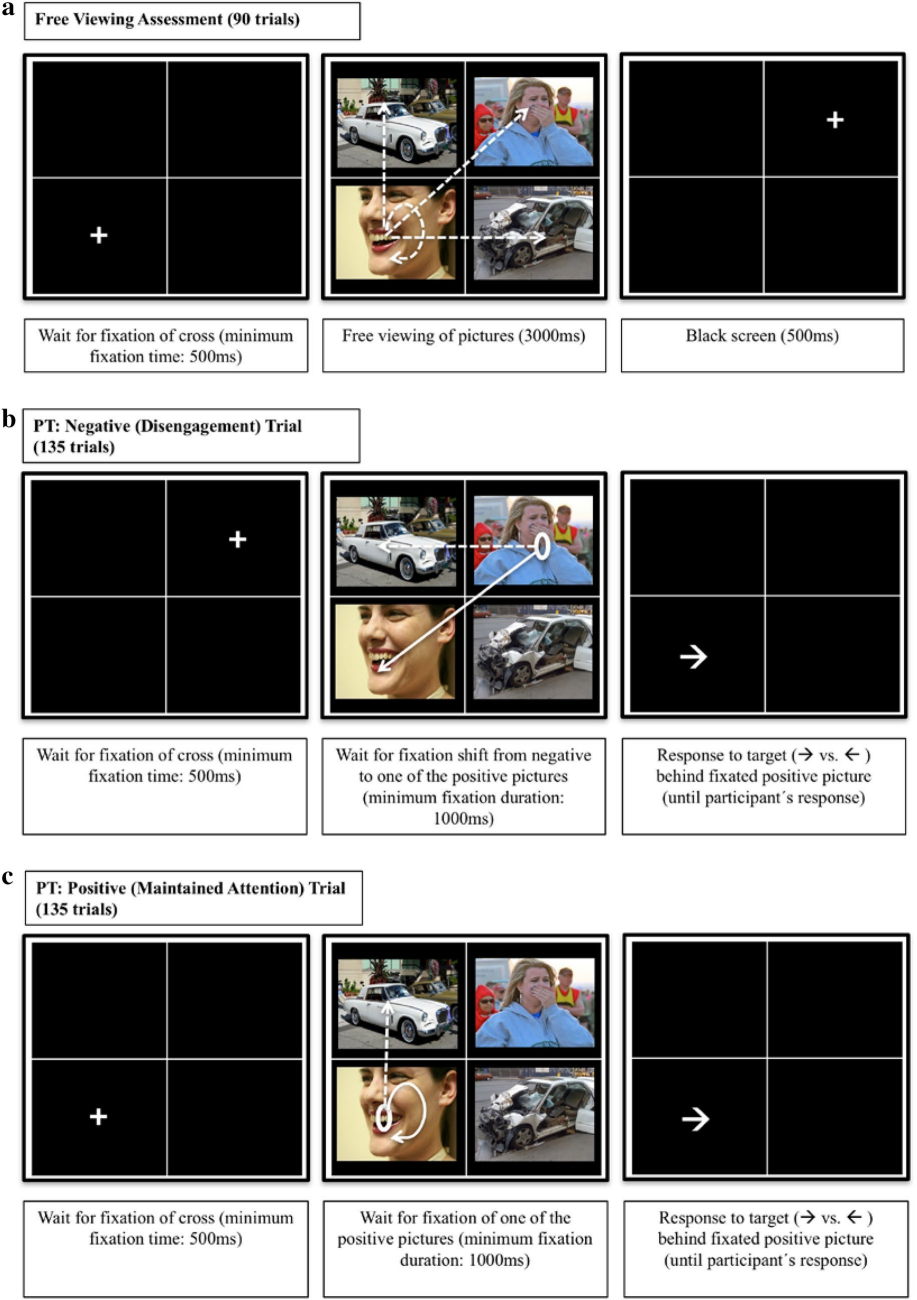

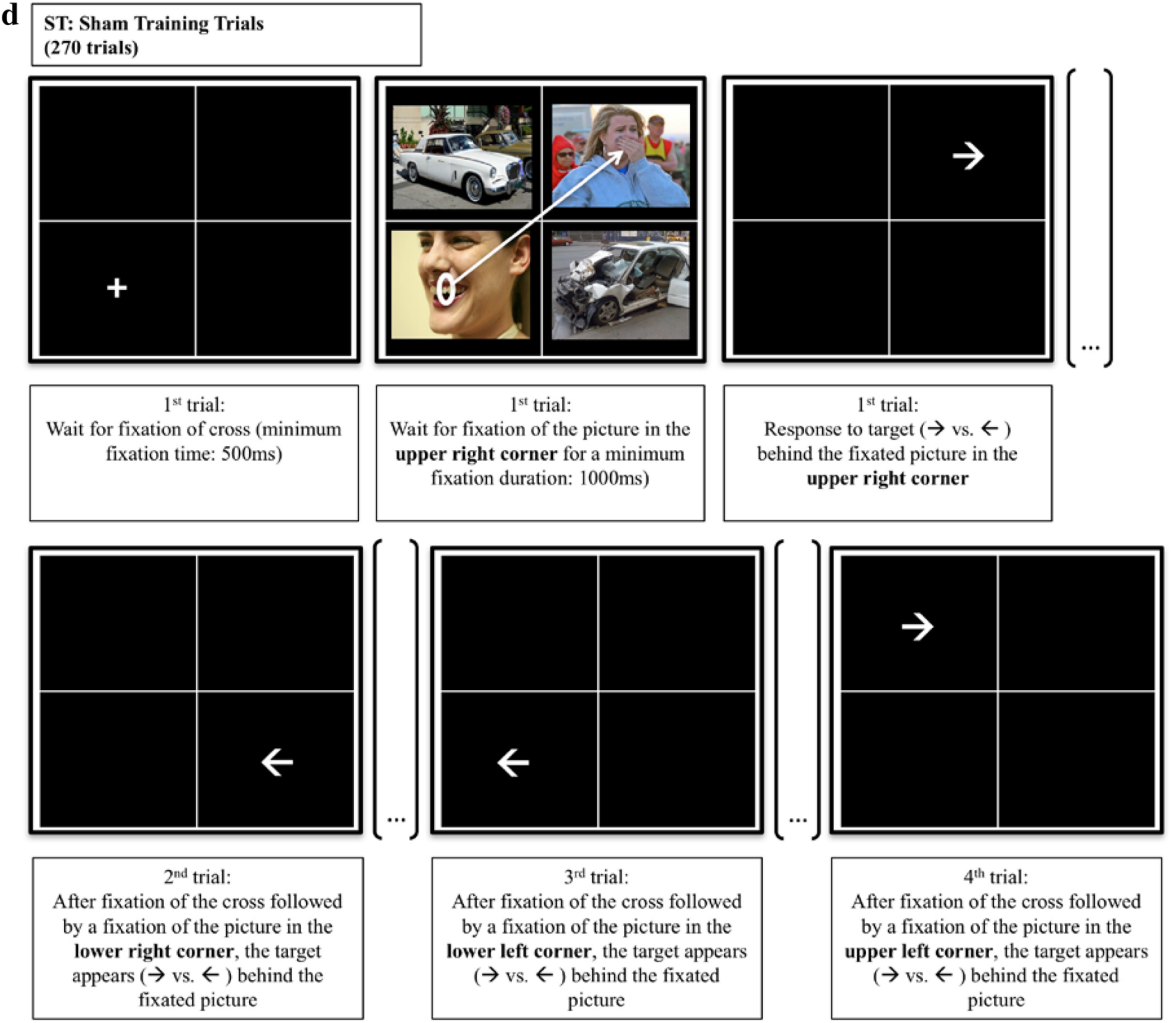
Schematic overview of the task design. On each trial of the positive training (PT), a fixation cross is presented. Upon fixation (500 ms), two negative and two positive pictures appear. **a** The free viewing task (assessment) is similar to the training, however, all trials last 3000 ms and no probe is presented. **b, c** Display a sample trial of the PT. **b** On negative (PT: disengagement) trials, participants have to disengage their attention from the fixated negative picture and fixate one of the two positive pictures. **c** On positive (PT: maintained attention) trials, attention has to be maintained at the fixated positive picture or at the other positive picture. **b, c** Upon fixation of a positive picture for 1000 ms, all pictures disappear and an arrow replaces the fixated picture. Participants respond to arrow direction by pressing a key. The arrow then disappears and a new trial starts. **d** An example of four consecutive trials in the sham training (ST), with the first trial requiring a fixation of a picture in the upper right corner. On each consecutive trial participants have to fixate a picture in a different corner, while the relevant corner changes in clockwise order (2nd trial: lower right corner; 3rd trial: lower left corner; 4th trial: upper left corner). Upon fixation of the picture in the correct corner for 1000 ms, all pictures disappear and an arrow replaces the fixated picture. Participants respond to arrow direction by pressing a key. The arrow then disappears and a new trial starts. *Note* This figure contains sample images, which have not been used in the current study. All images were obtained from Flickr and were published under a Creative Commons license. The formats of the images were slightly adapted for this figure. Credits: top left, Joe deSousa, CC0 1.0; top right, West Point—The U.S. Military Academy, CC BY 2.0; bottom left, Steven Depolo, CC BY 2.0; bottom right, bettyx1138, CC BY 2.0. For license terms see, CC0 1.0 (https://creativecommons.org/publicdomain/zero/1.0/); CC BY 2.0 (https://creativecommons.org/licenses/by/2.0/)

The eye-tracking task was adapted from Ferrari et al. (2016) and consisted of pre-assessment, training and post-assessment. For a graphic illustration of the task design, see Fig. 1. On each trial of the task, a white fixation-cross appeared in the middle of one of the four quadrants of the grid. As soon as the participant had fixated the cross for 500 ms, it disappeared and a set of four pictures was presented. Placing the fixation cross into one of the quadrants (instead of the screen center) and making sure that it was actually fixated, allowed for reliably manipulating which picture was fixated first.

The training contained two different types of trials: trials on which a negative picture replaced the cross (i.e., *negative* trials) and trials on which a positive picture replaced the cross (i.e., *positive* trials). Participants in the positive training (PT) had to disengage attention from negative pictures and shift it to positive pictures, and to maintain attention to positive pictures. On negative trials, participants had to look away from the fixated negative picture and fixate one of the two positive pictures for 1000 ms. Upon a sufficiently long fixation of a positive picture, all pictures disappeared and this previously fixated picture was replaced by a probe (i.e., an arrow pointing left or right, with the direction of the arrow being counterbalanced across picture valence). Participants had to identify the direction the arrow was pointing to by pressing a computer key, upon which the probe disappeared and a new trial started. On positive trials, the trial continued only if participants kept looking at the fixated positive picture for 1000 ms, or if they fixated the other positive picture in the picture set for 1000 ms.

In the sham training (ST), a different attentional pattern was trained, which was independent of the valence of the stimuli, such that neither disengagement from negative stimuli nor maintained attention to positive stimuli was selectively reinforced. Instead, participants were trained to show a clockwise viewing pattern of the presented picture sets. This means that, if on the first trial, the picture in the upper left part of the grid had to be fixated, the picture in the upper right part had to be fixated on the next trial, and the picture in the lower right part had to be fixated afterwards, and so on. The location of fixation cross, positive and negative pictures was counterbalanced, such that on negative (positive) trials, attention had to be disengaged from negative (positive) pictures as often as it had to be maintained to negative (positive) pictures. As in the PT, after a sufficiently long fixation of the correct picture, all pictures disappeared and a probe replaced the previously fixated picture. In both groups, the participants’ gaze pattern thus controlled the appearance of the probe. The training contained 270 training trials distributed across 3 blocks, during which each of the 45 picture sets was presented 6 times, in a new random order for each participant.

The pre- and post-assessment consisted of a free viewing task similar to the training and was introduced to participants as a calibration procedure. Different from the training, all picture sets were presented for 3000 ms, independently of participants’ viewing patterns, and no probe followed. During assessment, the 45 picture assessment sets were presented twice (90 trials), once as positive and once as negative trials. During each assessment-phase, the location of the fixation cross was counterbalanced across valences and grid positions. The entire task took approximately 45 min, depending on how quickly participants learned the required viewing patterns.

#### Eye-Tracking Device

Monocular gaze data of the dominant eye were obtained at a frequency of 500 Hz, by means of the iView X Hi Speed system by SMI, a video based eye-tracking system.

#### Calculation of Attentional Indices

In line with previous studies (Ferrari et al. 2016; Sanchez et al. 2013), only fixation durations of at least 100 ms were considered. About 1.5 percent of all trials at pre- and post-assessment were deleted, due to poor tracking quality or fixation durations shorter than 100 ms. The remaining trials were used to calculate three attentional indices separately for pre- and post-assessment: a “sustained attention bias” score, reflecting the proportion of the total fixation time on positive compared to negative pictures, as well as the two relevant attentional components, that is “disengagement from negative pictures” (short: negative disengagement) and “maintained attention to positive pictures” (short: positive maintained attention).

The sustained attention bias score was calculated in line with Ferrari et al. (2016): In the first step, we calculated two sum scores per trial, reflecting the total time participants fixated positive and negative pictures. Based on these scores, medians were calculated for each participant, representing the median fixation time on positive and negative pictures. In the last step, we calculated the bias score: (Median fixation time on positive pictures)/(median fixation time on positive pictures + median fixation time on negative pictures). Scores larger than 0.5 are indicative of a more positive sustained attention bias (relatively longer fixations on positive pictures), while scores smaller than 0.5 are indicative of a more negative sustained attention bias.

Negative disengagement scores and positive maintained attention scores were derived from negative and positive trials respectively. We calculated the median fixation duration on the first (positive or negative) picture until the first attentional shift to (and fixation of) one of the pictures of the opposite valence. Hence, on negative trials, longer fixation durations on negative pictures reflect a slower negative disengagement, while on positive trials, longer fixation durations on positive pictures reflect prolonged positive maintained attention.

#### Stress Task

In line with the study by Ferrari et al. (2016), we used an adapted speech task by Amir et al. (2008), in order to investigate training effects on emotional reactivity in response to a laboratory stressor. Via the computer, participants were informed that they would get 1 min to prepare a 3-min-speech on the topic “Why am I a good friend”. This topic has previously been used to assess the association between negative mood recovery after stress and attentional bias in depressed individuals (Sanchez et al. 2013). Participants were informed that their speech would be video-recorded so that it later can be evaluated on its quality by two independent researchers. To increase stress levels, participants were not allowed to take any notes during preparation and a clock on the computer screen signaled how much time was left. After 1 min, a “beep” sound occurred. The experimenter then entered the room, started the video-recording, asked participants to deliver their speech into a webcam, and left. After 3 min, the experimenter entered the room, stopped the video recording and left again for a 5-min resting phase. Participants were instructed to sit down quietly and relax. A clock on the screen again signaled the time left of the resting phase.

### Procedure

Prior to the experimental procedure, potential participants were pre-screened by means of the BDI via an online screening system of Radboud University Nijmegen. Only individuals with a score higher than 8 were invited for participation in the experiment, which took place within one week after pre-screening. These participants were tested individually in a cubicle of the Behavioural Science Institute of the University. After providing informed consent, they were randomly assigned in a double-blind fashion to one of the two training conditions (i.e., PT or ST). The experimenter then determined the participants’ dominant eye and subsequently calibrated the eye-tracker. Next, participants were seated in front of a computer, where they filled in the baseline questionnaires and mood state measures (T0: baseline). These measures were followed by a negative mood induction to re-activate the latent depressogenic schemas of the dysphoric sample (e.g., Beck 1967), before we assessed mood state a second time (T1: pre-training). Participants were then seated in front of the eye-tracker, where a brief calibration procedure was started, which was followed by pre-assessment, training and post-assessment of the ET-ABM task. If necessary, the calibration procedure was repeated before each training block and post-assessment. Afterwards, participants were again seated in front of the other computer, where the stress task was presented. Throughout this task, participants were asked to rate their mood state on the VAS scales: at the beginning of the task (T2: pre-stress), after the speech instructions were provided (T3: anticipatory stress), retrospectively during the speech (T4: during stress), after speech delivery (T5: post-stress), and after the 5-min resting period (T6: recovery). In addition to the VAS scales, we presented the Likert mood-scales at T2 (pre-stress), at T3 (anticipatory stress), and at T5 (post-stress). To assess possible training effects on state rumination, the MRSI was administered directly after the resting period. After having filled in the questionnaire, a sequence of a happy movie (Jungle book) was shown to elevate participants’ mood, followed by a final mood rating (VAS scale; T7). At the end of the experiment, participants filled in an awareness check, where we asked them to indicate on a 100 mm VAS scale, in how far they felt able to exert control on the eye-tracking task. Finally, participants were compensated for participation and debriefed. The entire procedure took about 120 min.

## Results

### Preliminary Analyses and Group Characteristics

In the analyses, we included only participants for whom both pre-assessment and post-assessment data of the eye-tracking task were available (*n* = 96). Of those, 13 participants had to be excluded from the analyses because of a BDI score lower than 9. Another 3 participants were excluded due to a lack of task adherence, and another 5 due to extreme values on the eye-tracking indices (i.e., data points more than 1.5 interquartile ranges below the first or above the third quartile). Due to skewness of the data, the attentional indices “negative disengagement” and “positive maintained attention” were log-transformed. Due to missing data of 23 participants on a single item of the Likert mood scales, we calculated means scores per participant instead of sum scores. The resulting groups (PT: *n* = 40, ST: *n* = 35), did not differ on any of the trait variables (*p* > .6) or mood state (*p* = .95) at baseline. Moreover, the groups did not differ on any of the demographic variables besides gender, with significantly more men in the PT (8 males) than in the ST (1 male), χ^2^(75) = 5.2, *p* = .023 (see Table 1). A manipulation check of the mood induction showed that VAS scores dropped from before to after the negative mood induction procedure, *F*(1, 72) = 140.04, *p* < .001, η^2^ = .66, similarly for both groups, *F*(1, 72) = .04, *p* = .839.

**Table 1:** Group differences on demographic variables and baseline questionnaires

	PT (*n* = 40)	ST (*n* = 35)	
Age	21.18 (2.36)	21 (2.76)	*t*(73) = 0.3, *p* = .771
Gender			*χ*^2^(1) = 5.2, *p* = .023
Male	8	1	
Female	32	34	
Nationality			*χ*^2^(1) = 0.14, *p* = .711
Dutch	20	19	
German	20	16	
BDI-II	17.53 (6.44)	16.64 (6.25)	*t*(73) = 0.73, *p* = .654
STAI-T	49.4 (9.33)	51.29 (9.82)	*t*(73) = 0.85, *p* = .952
RRS	2.3 (0.56)	2.14 (0.53)	*t*(71) = 1.29, *p* = .762

RRS scores of two participants were missing

*PT* positive training, *ST* sham training, *BDI-II* Becks Depression Inventory Second Edition, *STAI-T* Spielberger Trait Anxiety Inventory, *RRS* Ruminative Response Scale

### Attentional Processes at Baseline

#### Sustained Attention Bias

A univariate ANOVA of the sustained attention bias at the pre-assessment revealed no significant group difference, *F(*1, 73) = .54, *p* = .466. A subsequent one-sample t-test indicated that the bias score did not deviate significantly from zero, indicating that before the training, participants had neither a tendency to attend towards positive nor towards negative pictures, *t*(74) = 1.11, *p* = .271.

#### Positive Maintained Attention and Negative Disengagement

Two separate ANOVAs comparing the log-transformed attentional indices between the two groups, showed that PT and ST group did not differ from each other regarding the attentional components at baseline (positive maintained attention: *F*(1, 74) = 0.26, *p* = .612; negative disengagement: *F*(1, 74) = 1.71, *p* = .195). A subsequent paired-samples t-test revealed that at baseline, participants showed slower disengagement from negative pictures than from positive pictures, *t*(74) = 3.09, *p* = .003, *d* = .33. For descriptives, see Table 2.

**Table 2:** Mean fixation times (with standard deviations) in milliseconds during the free viewing task, and the resulting attentional bias scores

	PT		ST	
	Pre-training	Post-training	Pre-training	Post-training
Fixation time on positive pictures	1434 (270)	1797 (400)	1499 (273)	1658 (402)
Fixation time on negative pictures	1413 (301)	1000 (373)	1378 (281)	1189 (372)
Sustained attention bias score	0.51 (0.1)	0.64 (0.13)	0.52 (0.1)	0.58 (0.13)
Disengagement from negative pictures	711 (217)	555 (169)	643 (178)	745 (342)
Maintained attention for positive pictures	624 (202)	802 (531)	595 (160)	801 (408)

Sustained attention bias score: Proportion of fixation time on positive pictures compared to negative pictures; Disengagement from negative pictures: Latency of the first shift from a negative picture until fixation of a positive picture; Maintained attention for positive pictures: Latency of the first shift from a positive picture until fixation of a negative picture

*PT* positive training, *ST* sham training

### Training Effects on Attentional Processes

#### Changes in Sustained Attention Bias

A 2 (group: PT, ST) × 2 (time: pre, post) repeated-measures (RM) ANOVA of the sustained attention bias scores revealed a main effect of time, *F*(1, 73) = 92.18, *p* < .001, η^2^ = .56, which was moderated by the training, *F*(1, 73) = 14.64, *p* < .001, η^2^ = .17. As indicated by a subsequent paired-samples t-test, both groups showed an increase in sustained attention bias for positive pictures (PT: *t*(39) = 9.46, *p* < .001, *d* = 1.54; ST: *t* (34) = 4.21, *p* < .001, *d* = .76). The PT, however, showed a stronger bias after the training than the ST, *t*(73) = 2.83, *p* = .041, *d* = .48. For means, see Table 2.

#### Changes in Maintained Attention and Negative Disengagement

A 2 (group: PT, ST) × 2 (time: pre, post) × 2 (valence: positive, negative) RM ANOVA on the log-transformed attentional indices revealed a marginally significant effect of time, *F*(1, 73) = 3.12, *p* = .081, η^2^ = .04, which was moderated by valence, *F*(1, 73) = 27.17, *p* < .001, η^2^ = .27, as well as by group, *F*(1, 73) = 8.14, *p* = .005, η^2^ = .1. Importantly, the crucial 3-way interaction was significant as well, *F*(1, 73) = 8.94, *p* = .004, η^2^ = .11 indicating that the two groups showed differential changes in positive maintained attention and negative disengagement. Subsequent paired-samples t-tests revealed that participants in the PT learned to disengage more quickly from negative pictures, *t*(39) = 4.78, *p* < .001, *d* = .73, and to longer fixate positive pictures, *t*(39) = 2.11, *p* = .041, *d* = .36. Participants in the ST became generally slower with disengaging attention from both types of pictures (positive maintained attention: *t*(34) = 3.56, *p* = .001, *d* = .69; negative disengagement: *t*(34) = 2.13, *p* = .041, *d* = .36). While at pre-assessment, participants had the tendency to disengage from negative pictures more slowly than from positive pictures, this bias disappeared at post-assessment in the ST, *t*(34) = 0.47, *p* = .639, and was even reversed in the PT, *t*(39) = 3.48, *p* = .001, *d* = .57. See Table 2 for untransformed scores of the eye-tracking indices.

### Training Effects on Mood

#### Direct Effects on Mood

An independent-samples t-test on the VAS revealed that PT and ST did not differ in mood state right before the training, *t*(73) = 0.18, *p* = .859. A subsequent 2 (group: PT, ST) × 2 (time: pre-assessment, post-assessment) RM ANOVA of the VAS revealed a recovery from the negative mood induction procedure during the training, *F* (1, 72) = 106.96, *p* < .001, η^2^ = .6, which was not significantly different for the two groups, *F* (1, 72) = 1.3, *p* = .257. For means and SDs, see Table 3.

**Table 3:** Mean mood scores (with standard deviations) for all assessment points

	T0: baseline	T1: pre-training	T2: pre-stress	T3: anticipatory stress	T4: during stress	T5: post-stress	T6: recovery	T7: mood induction
PT								
VAS	56.85 (19.6)	27.95 (17.14)	56.63 (14.92)	48.24 (23.41)	46.76 (24.72)	56.42 (22.37)	55.71 (17.69)	68.18 (17.27)
Likert								
Happy	–	–	6.26 (1.48)	6 (1.84)	–	5.89 (1.62)	–	–
Anxious	–	–	3.64 (1.47)	4.66 (2.36)	–	3.88 (2.2)	–	–
Sad	–	–	3.58 (1.54)	3.86 (1.84)	–	3.4 (1.88)	–	
ST								
VAS	57.15 (21.33)	27.24 (17.13)	48.53 (16.87)	44.56 (22.71)	39.81 (25.95)	49.06 (20.06)	50.84 (16.34)	65.28 (18.86)
Likert								
Happy	–	–	5.42 (1.89)	5.39 (2.14)	–	5.41 (2.05)	–	–
Anxious	–	–	4.07 (2.14)	5.25 (2.36)	–	4.24 (2.47)	–	–
Sad	–	–	3.88 (1.84)	4.18 (2.28)	–	3.53 (2.11)	–	–

*PT* positive training, *ST* sham training, *VAS* Visual Analogue Scale of general mood state, *Likert* Likert mood scales = happy mood (items: happy, optimistic, joyful), anxious mood (items: nervous, tense, anxious), sad mood (items: depressed, upset, sad), *T1 pre-training* directly after the negative mood induction, *Pre-stress* directly after the training

### Effects on Mood Reactivity and Recovery in Response to Stress

#### VAS

A 2 (group: PT, ST) × 4 (time: pre-speech, announcement, during-speech, post-speech) RM ANOVA on the VAS, revealed a significant time effect, *F*(3, 66) = 9.7, *p* < .001, η^2^ = .31. Within-subject contrasts revealed that the speech task had its intended effects: The announcement of the task resulted in a drop in mood, *F*(1, 68) = 6.16, *p* = .016, η^2^ = .08, which remained low during the speech task, *F*(1,68) = 2.61, *p* = .111, while mood increased again after the speech task, *F*(1, 68) = 28.78, *p* < .001, η^2^ = .3. For means, see Table 3. However, the crucial time-by-group interaction was not significant, *F*(3, 66) = 0.46, *p* = .710, indicating that PT and ST did not differentially affect mood reactivity or recovery in response to the stress task.

#### Likert Scales

The 2 (group: PT, ST) × 4 (time: pre-speech, announcement, during-speech, post-speech) RM ANOVA was repeated for each of the three Likert scales (i.e., happiness, anxiety and sadness). While happiness ratings remained unaffected by the speech task, *F*(2, 67) = 0.7 *p* = .449, anxiety and sadness ratings changed throughout the speech task (Anxiety: *F*(2,67) = 14.25, *p* < .001, η^2^ = .3; Sadness: *F*(2,67) = 10.03, *p* < .001, η^2^ = .23). Inspection of the means suggests that anxiety and sadness increased in response to the announcement (Anxiety: T2 *M* = 3.84 (*SD* = 1.8), T3 *M* = 4.93 (*SD* = 2.36); Sadness: T2 *M* = 3.72 (*SD* = 1.68), T3 *M* = 4.01 (*SD* = 2.05)), and dropped again after the speech task (Anxiety: T5 *M* = 4.05 (*SD* = 2.32); Sadness T5 *M* = 3.46 (*SD* = 1.97)). However, in line with the analysis of the VAS, the crucial interaction effect was not significant, suggesting that changes in mood in response to the speech task did not differ between groups (Anxiety: *F*(2, 67) = 0.19, *p* = .827; Sadness: *F*(2, 67) = 0.32, *p* = .73).

### Training Effects on State-Rumination

We compared MRSI scores after the stress task between the two groups by means of an independent-samples t-test. This analysis revealed no significant training effect on state rumination (PT: *M* = 20.32 (*SD* = 6.85); ST: *M* = 21.91 (*SD* = 6.69); *t*(86) = 0.98, *p* = .332).

### Contingency Awareness

The awareness check revealed that relatively more participants in the PT became aware of the training contingency than in the ST, 55% versus 12%; χ^2^(74) = 15.07, *p* < .001. Moreover, participants in the PT reported more perceived control over the eye-tracking task than participants in the ST, *t*(73) = 3.98, *p* < .001. Therefore, we repeated the main analysis with awareness as an additional between-subjects factor. The 2 (group: PT, ST) × 2 (time: pre, post) × 2 (contingency awareness: yes, no) RM ANOVA of the sustained attention bias revealed no 3-way interaction effect involving awareness, *F*(1, 70) = 2.66, *p* = .107. The 2 (group: PT, ST) × 2 (time: pre, post) × 2 (valence: positive, negative) × 2 (contingency awareness: yes, no) RM ANOVA on the log-transformed attention bias indices did not indicate a significant 4-way interaction with awareness either, *F*(1,70) = 3.36, *p* = .071.

## Discussion

To address the limitations of previously used RT-based ABM paradigms, a novel ABM paradigm based on eye-tracking was recently developed (i.e., the ET-ABM; Ferrari et al. 2016). This paradigm was specifically designed to assess and target the attentional components that are biased in depression: the disengagement from negative stimuli and the maintained attention to positive stimuli. A first proof-of principle study with healthy students showed that, compared to a negative training version, the ET-ABM can induce a positive sustained attention bias as well as faster disengagement from negative stimuli. The aim of the present study was to replicate these promising findings in an emotionally vulnerable sample of dysphoric students, with a placebo sham-training as control condition.

In line with the findings of Ferrari et al. (2016), the PT induced a positive sustained attention bias (i.e., longer fixations on positive than on negative stimuli). Notably, both PT and ST showed an increase in positive sustained attention bias. However, this increase was stronger in the PT group, supporting the effectiveness of the training in modifying attentional processes in dysphoric individuals. As in the previous study, these general training effects were again driven by reduced disengagement latencies from negative stimuli in the PT group. Beyond that, the PT group also showed an increase in maintained attention to the first fixated positive pictures, suggesting that the training may also affect the initial processing of positive stimuli. In contrast, the ST did not induce any valence-specific attentional viewing patterns. Instead, participants in this group became generally slower with directing their gaze away from the initially fixated pictures, resulting in slower disengagement from both negative and positive pictures. Summarizing, while the PT was effective in reversing an initially negative attentional bias into a positive attentional bias, characterized by relatively longer fixations on positive pictures and by relatively quicker disengagement from negative pictures, the ST resulted in a decline of the negative bias. A potential explanation for the observed effect in the ST group might be that the ST was possibly more difficult than the PT. Remarkably, more participants in the PT became aware of the reinforced training pattern, and the PT group experienced a stronger feeling of control over the task. It is likely that the ST therefore was more tiring than the PT, resulting in slower latencies in general.

In a previous study (Ferrari et al. 2016), the PT did not modify initial maintained attention to positive pictures. It had been suggested that the temporal criteria defining a fixation as sufficiently long to continue a training trial (i.e., 1000 ms) might not be appropriate to induce “longer” maintained attention to positive stimuli. As our main goal was to replicate the earlier training effects on general sustained attention and attentional disengagement from negative stimuli, we did not increase the required fixation duration on positive pictures. Nevertheless, in the current study, training effects were partially driven by the increased initial maintained attention to positive stimuli. This might be explained by the different stimulus sets used in the two studies. In the previous study, negative and positive pictures were matched on their valence ratings, whereas this was not done in the current study. As a result, more extreme positive and negative pictures might have been presented during the training, which possibly resulted in a better contrast between these valences. This change in contrast might have helped participants to more easily identify the two different responses required to react to the two different picture types, resulting in the modification of both indices, disengagement from negative pictures and maintained attention to positive pictures. Although this explanation remains speculative, the current findings provide promising evidence that the ET-ABM may actually directly tap into both components of attention that are relevant in depression. Importantly, as different picture sets were used during training and assessments, we may further conclude that the observed training effects are not merely the result of stimulus-specific response patterns, but reflect a modified attentional processing of emotionally valenced information in general.

To get a first indication of the potential therapeutic effects of the ET-ABM, we additionally explored changes in mood. In contrast to the first study, the PT and ST did not differentially affect participants’ mood state. In general, positive mood increased indistinguishably in both groups from before to after the training, possibly pointing to recovery from the negative mood induction at the beginning of the experiment. More importantly, the training did not affect mood changes during the stress task either. In line with the previous study (Ferrari et al. 2016), PT and ST groups did not differ in their mood reactivity or recovery from the speech challenge. It was therefore speculated that stress-attenuating effects of the training may be restricted to emotionally vulnerable samples, as suggested by previous CBM research (Becker et al. 2016). Our sample did consist of individuals with elevated depression scores. Hence, one possible interpretation of our results may be that in depression, the modification of attentional processes does not affect mood reactivity or recovery.

In the context of anxiety, the link between attentional bias and emotional vulnerability has been investigated in a range of studies (for a review, see Clarke et al. 2014), whereas only a few studies have addressed this topic in depression. Although Sanchez et al. (2013) did not experimentally manipulate attentional processes, they found that slower disengagement from negative stimuli predicted lower mood recovery after stress. Together with the few studies showing that reducing a negative attentional bias may attenuate depressive symptoms (Browning et al. 2012; Wells and Beevers 2010; Yang et al. 2014), this provides evidence supporting a causal link between attentional bias and maintenance of depressed mood. It is important to note here, that the latter studies all made use of multiple training sessions, distributed over a longer period of time. However, given the limited number of studies and the contradicting conclusions from meta-analyses regarding number of sessions as a moderator of training effects (Beard et al. 2012; Cristea et al. 2015; Hallion and Ruscio 2011; Mogoaşe et al. 2014), it remains to be investigated whether training effects on stress reactivity and recovery, or even depressive symptoms, can be achieved by increasing the number of training sessions.

Moreover, we would like to emphasize that the stress task employed in this study may not be optimal for measuring transfer effects of the training to mood responses, which might be the reason why we found no relation between attentional processing and emotional reactivity. Although Sanchez et al. (2013) found a significant association of slow disengagement from negative stimuli with lower mood recovery after a speech challenge as used in our study, one may question whether a performance-related speech-challenge can be considered a relevant stressor in the context of depression. In fact, the link with attentional processes was exclusively found for “sad” mood recovery. Hence, before drawing firm conclusions about the causal role of attentional bias in depressed mood, future research should consider to increase the number of training sessions and investigate its effect on mood recovery after a depression-relevant stressor. Such a stressor could for instance involve a video-clip that induces sad mood, as used at the beginning of our experiment.

Subsequent studies using this paradigm might also want to include other measurement instruments that can detect far transfer effects. Even though this study administered a free-viewing assessment task with different pictures than used during training, the free-viewing task shares several characteristics with the training paradigm. To rule out that only a single task-relevant component has been trained, we recommend to make additional use of alternative bias measures, such as a spatial-cueing task (Baert et al. 2010) or the engagement-disengagement eye-tracking task by Sanchez et al. (2013). Moreover, as the current study does not allow to attribute training effects to either of the two attentional components (i.e., negative disengagement or positive maintained attention), follow-up studies are required to disentangle the specific working mechanism of the ET-ABM. Finally, a measurement-only control condition might be a useful addition for future research, as sham-training procedures as implemented in this study may have training unspecific effects as well (e.g., Gladwin 2017; Wells and Beevers 2010).

For these follow-up studies, we would strongly recommend to take all three attentional components into account (i.e., sustained attention, negative disengagement, positive maintained attention). The current study found no sustained attention bias at baseline, which might have been expected based on the extensive literature on the existence of negative attentional biases in depression (e.g., Peckham et al. 2010). However, the fact that we found a slower disengagement from negative than from positive pictures points to the importance of looking into the different attentional components separately, rather than exclusively investigating a general bias. Differentiating between negative disengagement and positive maintained attention may allow for a more sensitive measurement of attentional bias in depression.

To summarize, this study provides further support for the effectiveness of the ET-ABM in modifying the specific attentional components assumed to be causally involved in the development and maintenance of depression. In order to investigate whether ABM can indeed alleviate emotional vulnerability, it has been suggested that we need refined or new paradigms which reliably assess and modify these processes (Clarke et al. 2014). This study suggests that the ET-ABM task may indeed be such a paradigm that facilitates further progress in this field of research. Fortunately, the technical developments of the past decade made eye-tracking devices accessible at reasonable prices and suitable for the use in hospitals without requiring the expertise of technicians. Therefore, the next step should be to investigate the beneficial effects of the ET-ABM on clinically relevant measures in a patient sample.
